# Association of Social Isolation of Long-term Care Facilities in the United States With 30-Day Mortality

**DOI:** 10.1001/jamanetworkopen.2021.13361

**Published:** 2021-06-16

**Authors:** Chanhyun Park, Daniel Kim, Becky A. Briesacher

**Affiliations:** 1 Department of Pharmacy and Health Systems Sciences, Bouvé College of Health Sciences, Northeastern University, Boston, Massachusetts; 2 Department of Health Sciences, Bouvé College of Health Sciences, Northeastern University, Boston, Massachusetts; 3 School of Public Policy and Urban Affairs, Northeastern University, Boston, Massachusetts

## Abstract

**Importance:**

Long-term care (LTC) residents may be susceptible to social isolation if living in facilities located in neighborhoods lacking social connection.

**Objective:**

To characterize the social isolation of residents living in LTC facilities in the US.

**Design, Setting, and Participants:**

This cross-sectional study included 730 524 LTC residents from 14 224 LTC facilities in 8652 zip code tabulation areas (ZCTAs) in the US in 2011. A nationwide LTC database with ZCTA data was linked to population-level geographic data from the US Census Bureau. Statistical analysis was performed from January 2019 to December 2020.

**Exposures:**

The primary variable of interest was the social isolation of LTC neighborhoods defined as the percentage of households in the ZCTA with individuals aged 65 years or older who lived alone and categorized into quartiles of social isolation.

**Main Outcomes and Measures:**

Maps were generated to illustrate geographic variation of LTC facilities at the ZCTA level by the quartile of socially isolated neighborhoods. Generalized estimating equations were used to estimate the adjusted likelihood that LTC facilities were located in areas of highest social isolation. We also used multilevel logistic regression models to assess the association between the social isolation of neighborhoods of LTC facilities and 30-day all-cause mortality after LTC admission. Subgroup analyses were conducted by race and ethnicity.

**Results:**

Among 33 120 ZCTAs in the US, 8652 (26.1%) had at least 1 LTC facility. Among the 730 524 LTC residents included in the study’s 14 224 LTC facilities, 458 136 (62.71%) were female, 610 802 (83.61%) were non-Hispanic White, and 419 654 (57.45%) were aged 80 years or older. Location of LTC facilities was associated with increasing levels of social isolation (quartile 1 = 9.72% [n = 840]; quartile 2 = 18.60% [n = 1607]; quartile 3 = 32.23% [n = 2784]; quartile 4 = 39.45% [n = 3408]; *P* < .001). In multivariate models, LTC facilities were 8 times more likely to be located in ZCTAs with the highest percentages of older adults residing in single-occupancy households (odds ratio [OR], 8.46; 95% CI, 7.44-9.65; *P* < .001), compared with ZCTAs with the lowest percentages. This association held across ZCTAs with a majority population of African American and Hispanic individuals, although it was strongest in ZCTAs with a majority population of White individuals. LTC residents entering facilities in neighborhoods with the highest levels of social isolation among older adults had a 17% higher risk of 30-day mortality (OR, 1.17; 95% CI, 1.10-1.25; *P* < .001) compared with those in neighborhoods with the lowest levels of social isolation among older adults.

**Conclusions and Relevance:**

This study found that LTC facilities were often located in socially isolated neighborhoods, suggesting the need for special attention and strategies to keep LTC residents connected to their family and friends for optimal health.

## Key Points

**Question:**

To what extent are US long-term care facilities located in socially isolated neighborhoods and what is the association with the risk of 30-day mortality?

**Findings:**

This cross-sectional study found that long-term care facilities in the US were approximately 8 times more likely to be located in neighborhoods with the highest percentage of individuals aged 65 years or older living alone compared with neighborhoods with the lowest percentage. Long-term care facilities in socially isolated neighborhoods were associated with increased risk of 30-day all-cause mortality among residents.

**Meaning:**

The findings suggest the need for special attention and strategies to keep long-term care residents connected to their family and friends.

## Teaser

This cross-sectional study examines the prevalence of long-term care (LTC) facilities in socially isolated neighborhoods in the US and assesses the 30-day mortality risk associated with residence in an LTC facility located in an area with a high level of social isolation.

## Introduction

In mid-March of 2020, long-term care (LTC) facilities were required by the Centers for Medicare and Medicaid Services to ban all nonessential visitors and group activities in response to COVID-19 safety rules. Within a few months of the lockdown, LTC staff and clinicians began reporting increased levels of depression, anxiety, worsening dementia, and failure to thrive in residents.^1^ These accounts have drawn attention to the role of social isolation in LTC facilities.

Social isolation—generally defined as having few social network ties or infrequent social contact—is an important public health concern that affects many older adults.^2^ Living alone or living in neighborhoods with a high proportion of single person households have been found to be predisposing factors to social isolation.^3^ In the community setting, 28% (14.7 million) of all older adults aged 65 years or older live alone (5.0 million men, 9.7 million women).^4^ By age 85, 39% of older adults live alone.^5^ Socially isolated adults experience high rates of negative health outcomes, including premature mortality and a 50% increase in the risk of dementia.^6,7^ Living alone is a strong risk factor for LTC placement.^8,9^ Growing evidence suggests differences in social isolation by race/ethnicity,^10^ although an association between mortality risk and social isolation has been found in both non-Hispanic White individuals and African American individuals.^11^

In the LTC setting, social isolation is less well defined and rarely studied. By definition, LTC residents live in a facility with others so social isolation often refers to the loss of personal connection to family and friends outside the facility. Concern about social isolation is important to LTC residents: proximity to prior residence is the strongest factor associated with the choice of LTC facility.^12^ Socially isolated LTC residents may be at higher risk of negative outcomes. In a study of 323 LTC residents with advanced dementia, 88% received outside visitors for a least 1 hour a week, but 12% never received any visitors during an 18-month period.^13^ Among the LTC residents who had no visitors, reports were higher of pain, pressure ulcers, and dyspnea compared with residents with regular weekly visitors. At least 1 study has found evidence of shorter survival time among LTC residents who were admitted to facilities located in socially isolated areas at the county level.^14^

To our knowledge, there is no information on the extent that LTC facilities are located in socially isolated neighborhoods. The objectives of this study were to characterize the social isolation of LTC facilities in the US and to assess short-term (30-day) all-cause mortality risk in residents within the LTC facilities that experience the social isolation. We posit that the social isolation of LTC facilities may be important if it is associated with barriers to connections with friends and family and negative health risks to their residents that have been documented in the community setting.

## Methods

### Data Sources

We linked the following data sets: (1) 2011 Certification and Survey Provider Enhanced Reporting (CASPER), (2) 2011 American Community Survey (ACS) 5-year estimates, (3) 2010 US Census, (4) 2011 3.0 Minimum Data Set (MDS), (5) 2011 Master Beneficiary Summary file, and (6) 2011 Medicare Parts A and B claims data.

Data detailing the location of LTC facilities were extracted from the CASPER data set. CASPER is a repository of the validated, federally mandated, on-site surveys of all Medicare- and Medicaid-certified long-term care facilities in the US. The LTC surveys are conducted by state survey agencies every 15 months at minimum, or in the event that a complaint is filed. CASPER data include information about the facility operational characteristics and aggregate patient characteristics. The reliability and validity of the CASPER data have been demonstrated previously.^15,16,17^

Data on the zip code Tabulation Area (ZCTA)-level percentage of older adult residents who were aged 65 and older and living alone came from the decennial 2010 US Census.^18^ All other ZCTA-level aggregate data on demographic and socioeconomic characteristics were drawn from the 5-year combined 2009 to 2013 ACS, centered in 2011.^19^ The ACS and US Census data were abstracted using American Fact Finder from the US Census Bureau.^20^

The MDS is a federally mandated clinical assessment of residents living in LTC facilities that are certified by Medicare or Medicaid. The MDS data contain information about active diagnoses, psychosocial well-being, and physical functioning at admission, quarterly intervals, and when there are changes in health status. The reliability and validity of the MDS data have been demonstrated previously.^21,22^

Medicare data came from the Medicare program and included enrollment information, including the date of death. The Medicare Parts A and B claims data included information about diagnoses and health care utilization.

Because all data used in this study were deidentified, this study was approved and classified as exempt by the Northeastern University institutional review board and informed consent was waived, in accordance with 45 CFR §46. This study followed the Strengthening the Reporting of Observational Studies in Epidemiology (STROBE) reporting guideline for cross-sectional studies.

### Study Population

Out of a total of 33 120 ZCTAs in the US, 8652 ZCTAs had at least 1 LTC facility. This cross-sectional study included 14 224 LTC facilities and 730 524 LTC residents from these 8652 ZCTAs in 2011. Detailed information about the characteristics of LTC residents was reported in a previous study.^14^

### Measures

#### Social Isolation of the ZCTAs

Our primary exposure variable of interest was the social isolation in ZCTA. The degree of social isolation is defined as the percentage of households in the ZCTA with individuals aged 65 years or older who lived alone.^23^ The degree of social isolation was calculated by dividing the number of individuals aged 65 years or older living alone by the number of households with individuals aged 65 years or older in the ZCTA. These results were then categorized into quartiles: quartile 1, <30.77% (the lowest social isolation); quartile 2, 30.77% to 36.69%; quartile 3, 36.70% to 42.39%; and quartile 4, >42.39% (the highest social isolation) of households in the ZCTA with individuals 65 years or older who lived alone.

#### Presence of LTCs in ZCTAs

To estimate whether LTCs were located in areas with the highest levels of social isolation for older adults, the binary dependent variable was having at least 1 LTC facility in the ZCTA area at the ZCTA level. The zip codes of LTC facilities were obtained from the CASPER data and then linked with ZCTA information from US Census data.

#### 30-Day All-Cause Mortality

To assess whether 30-day all-cause mortality was higher in areas of highest social isolation, the binary dependent variable was 30-day all-cause mortality after admission to an LTC facility at the individual level. We obtained 30-day all-cause mortality of both short-term (<100 days) and long-term (≥100 days) LTC residents.

#### Covariates

We included 3 levels of covariates: individual, LTC facility, and ZCTA levels. ZCTA-level covariates were used to estimate whether LTC facilities were located in areas of highest social isolation. Individual, LTC facility, and ZCTA covariates were used to assess whether 30-day mortality after admission to LTC facility was higher in areas of highest social isolation.

We controlled for individual-level covariates that consisted of age groups (aged ≤64 years, 65-79 years, ≥80 years), sex (male, female), race/ethnicity (White, African American, Hispanic, other races), marital status (married, never married, widowed, separated, divorced), length of LTC stay (<100 days, ≥100 days), These variables were extracted using the MDS data. In addition, we included Medicaid enrollment and comorbid conditions using the validated Prescription Drug Hierarchical Condition Category index.

We used the self-reported race/ethnicity measure contained in the Medicare administrative data and collected as part of federally mandated long-term care assessments. We measured race and ethnicity in this study to assess any disparities in social isolation of LTC facilities.

At the LTC facility level, we controlled for the percentage of LTC residents who were aged 65 years and older, the percentage of female LTC residents, the percentage of LTC residents by various races (eg, White, African American, Hispanic, other races), and the percentage of LTC residents by marital status (married, never married, widowed, separated, divorced). These variables were extracted using the MDS data. In addition, we included the percentage of LTC residents with Medicaid enrollment, and whether the LTC facility belonged to a chain and was a profit vs nonprofit institution.

At the ZCTA level, using 5-year average data from the ACS data, we included the following ZCTA-level covariates: the percentage of adults who were aged 65 years and older, the percentage of female residents, the percentage of races (eg, White, African American, Asian, Hispanic, other races), the percentage of education levels, the percentage of married, the percentage of owner-occupied housing, the percentage of Medicaid enrollment, the urbanicity of area, and the 9 US Census divisions.

### Statistical Analyses

First, we examined the unadjusted differences in area characteristics between the ZCTAs with and without LTC facilities using a *t* test for continuous covariates and a χ^2^ test for categorical variables. Second, we examined the generalized estimating equations model with logit link to estimate the association between the percentage of households with individuals aged 65 years or older who lived alone and the presence of nursing homes within that ZCTA. We estimated adjusted odds ratios (ORs) and 95% CIs of having LTC facilities by the quartile of socially isolated neighborhoods at the ZCTA level, controlling for covariates. We also conducted subgroup analyses in ZCTAs with a majority population of White, African American, or Hispanic populations as defined by US Census percentages above the national median. All model standard errors were adjusted by clustering within the ZCTA.

Third, using multilevel logistic regression models, we estimated adjusted ORs and 95% CIs of individual risk of 30-day morality from any cause by the quartile of socially isolated neighborhoods, controlling for covariates. We further performed subgroup analyses by racial and Hispanic ethnicity populations: (1) non-Hispanic White individuals; (2) non-Hispanic African American individuals; and (3) Hispanic individuals. All models were estimated using Stata version 16.0 (StataCorp).

Finally, we created maps to visualize the presence and absence of LTC facilities at the ZCTA level by the quartile of socially isolated neighborhoods. We used ArcGIS Pro version 2.5.1 (Environmental Systems Research Institute) to generate the maps of the US, and the zip Code Tabulation Areas Shapefile was used to enable ArcGIS Pro to create the ZCTA-level maps.^24^ Using ArcGIS Pro*,* the Query Builder tool and Select Feature by Attribute tool have been used to query ZCTA codes with presence and absence of LTC facilities in each ZCTA. Both tools can be used to build a Structured Query Language query to create a selection based on map attribute. Through the Categorized Symbology tool, specific colors had been assigned to different features, which were the quartiles of socially isolated neighborhoods. All statistical tests were 2-sided with a significance level of .05.

## Results

Among 33 120 total ZCTAs in the US, 8652 (26.1%) had at least 1 LTC facility. We included 730 524 LTC residents in 14 224 LTC facilities; 458 136 (62.71%) were female, 610 802 (83.61%) were non-Hispanic White, and 419 654 (57.45%) were aged 80 years or older. Table 1 describes the area characteristics between the neighborhoods with LTC facilities and those without LTC facilities. Location of LTCs was associated with increasing levels of social isolation (Q1 = 9.72% [n = 840], Q2 = 18.60% [n = 1607], Q3 = 32.23% [n = 2784], Q4 = 39.45% [n = 3408], *P* < .001). Detailed baseline demographic characteristics of these LTC residents was reported in a previous study.^14^

**Table 1.** Area Characteristics of ZCTAs With and Without Long-term Care Facilities

Characteristic	ZCTA			*P* value
	With LTC	Without LTC	Overall	
No.	8652	24 468	33 120	
Age, ≥65 y, mean (SD), %	15.61 (6.38)	16.12 (11.11)	15.99 (10.08)	<.001
Sex, mean (SD), %				
Female	51.00 (3.02)	49.59 (6.75)	49.97 (6.03)	<.001
Race, mean (SD), %				
White	79.94 (20.53)	85.73 (20.68)	84.20 (20.80)	<.001
African American	10.59 (17.55)	6.54 (15.33)	7.61 (16.05)	<.001
Asian	2.91 (5.79)	1.56 (4.85)	1.91 (5.15)	<.001
Others	6.56 (8.55)	6.17 (12.8)	6.27 (11.88)	.008
Ethnicity, mean (SD), %				
Hispanic	10.79 (16.30)	7.68 (16.23)	8.50 (16.30)	<.001
Education, mean (SD), %				
Less than high school	14.73 (8.80)	15.37 (12.12)	15.19 (11.38)	<.001
High school	31.50 (9.59)	35.71 (14.08)	34.60 (13.18)	<.001
Any college or higher	53.77 (14.72)	48.92 (18.62)	50.20 (17.80)	<.001
Married, mean (SD), %	50.72 (10.40)	55.11 (15.45)	53.96 (14.42)	<.001
House owner, mean (SD), %	68.43 (14.66)	76.66 (17.71)	74.48 (17.34)	<.001
Medicaid enrollment, mean (SD), %	14.60 (8.52)	13.48 (14.06)	13.77 (12.86)	<.001
Median household income, mean (SD), $	51 971 (20 286)	51 022 (22 585)	51 277 (21 996)	<.001
Population size, No. (%)				
<1000	174 (2.01)	9939 (40.62)	10 113 (30.53)	<.001
1000-9999	2863 (33.09)	10 592 (43.29)	13 455 (40.63)	
≥10 000	5615 (64.90)	3937 (16.09)	9552 (28.84)	
Area, No. (%)				
Urban	3313 (38.29)	4315 (17.64)	7628 (23.03)	<.001
Rural	5339 (61.71)	20 153 (82.36)	25 492 (76.97)	
Census division, No. (%)				
New England	493 (5.70)	1338 (5.47)	1831 (5.53)	<.001
Middle Atlantic	956 (11.05)	3231 (13.21)	4187 (12.64)	
East North Central	1597 (18.46)	3519 (14.38)	5116 (15.45)	
West North Central	1357 (15.68)	3519 (14.38)	4876 (14.72)	
South Atlantic	1256 (14.52)	3884 (15.87)	5140 (15.52)	
East South Central	615 (7.11)	1847 (7.55)	2462 (7.43)	
West South Central	1159 (13.40)	2530 (10.34)	3689 (11.14)	
Mountain	422 (4.88)	2156 (8.81)	2578 (7.78)	
Pacific	797 (9.21)	2444 (9.99)	3241 (9.79)	
Older single-occupancy household, No. (%)^a^				
Quartile 1: <30.77%	840 (9.72)	7351 (30.58)	8191 (25.07)	<.001
Quartile 2: 30.77%-36.69%	1607 (18.60)	6541 (27.21)	8148 (24.93)	
Quartile 3: 36.70%-42.39%	2784 (32.23)	5386 (22.41)	8170 (25.00)	
Quartile 4: >42.39%	3408 (39.45)	4761 (19.81)	8169 (25.00)	

Abbreviations: LTC, long-term care; ZCTA, zip code tabulation area.

^a^ Households with a single resident aged 65 years or older. The number of ZCTAs with a missing age value was 442.

**Figure 1.**
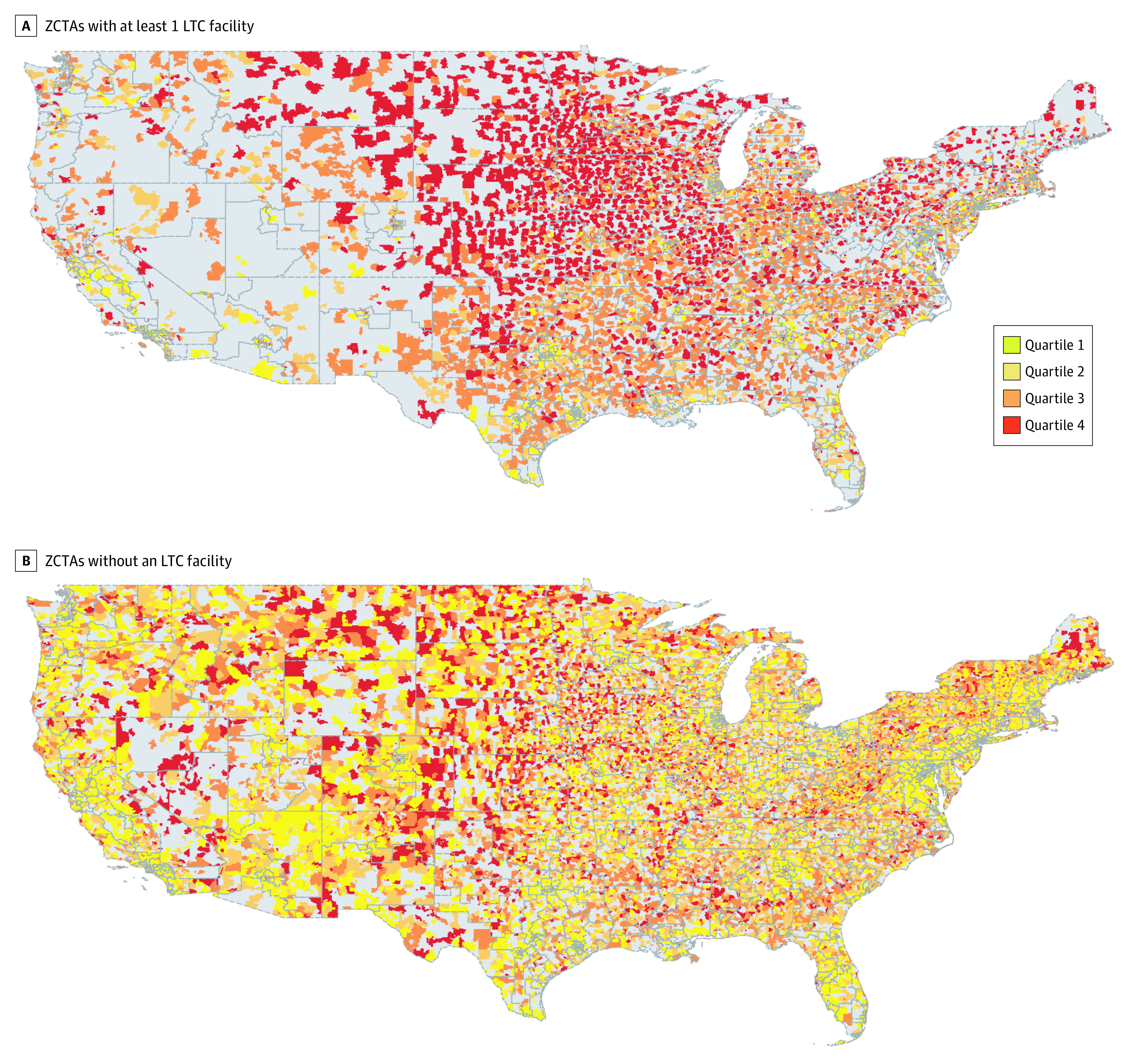
Zip Code Tabulation Areas (ZCTAs) With and Without Long-term Care Facilities (LTC) by the Quartile of Older Single-Occupancy Household A, the first map was mostly red, which indicates that LTC facilities were mostly in areas with the highest levels of social isolation of older adults. B, the second map was mostly yellow, which indicates that areas of low social isolation of older adults do not usually have LTC facilities.

The association between socially isolated neighborhoods and the location of LTC facilities is visualized in Figure 1. Figure 1A displays the presence of at least 1 LTC facility by the 4 levels of social isolation across the contiguous US map, while Figure 1B shows areas without LTC facilities. We see the first map was mostly red, which indicates that LTC facilities were mostly in areas with the highest levels of social isolation of older adults. In contrast, the second map was mostly yellow, which indicates that areas of low social isolation of older adults do not usually have LTC facilities. Most of the socially isolated LTC facilities are in the Midwest section of the US (as seen in Figure 1A).

Table 2 shows the results of the models estimating the likelihood of having LTC facilities in the ZCTA, adjusted for ZCTA-level covariates. The most socially isolated neighborhoods were significantly more likely to have LTC facilities than less socially isolated neighborhoods. The odds of having LTC facilities were approximately 8 times higher in ZCTAs falling in the highest quartile of older adults’ single-occupancy households (OR, 8.48; 95% CI, 7.44 to 9.65; *P* < .001), approximately 5 times higher in ZCTAs in the third quartile (OR, 4.98; 95% CI, 4.44 to 5.58; *P* < .001), and approximately 2 times higher in ZCTAs in the second quartile (OR, 2.33; 95% CI, 2.09 to 2.61; *P* < .001) compared with those in the lowest quartile.

**Table 2.** Odds Ratio of Having Long-term Care Facilities in Zip Code Tabulation Area^a^

Variable^b^	OR (95% CI)	*P* value
Age, ≥65 y	1.060 (1.054-1.065)	<.001
Female	1.040 (1.031-1.050)	<.001
Race		
White	0.994 (0.990-0.999)	.02
African American	0.996 (0.991-1.001)	.10
Asian	1.006 (0.998-1.015)	.16
Ethnicity		
Hispanic	1.005 (1.002-1.008)	.003
Education		
Less than high school	1.010 (1.004-1.015)	.001
High school	1.002 (0.998-1.007)	.35
Married	0.993 (0.988-0.998)	.009
House owner	0.987 (0.983-0.990)	<.001
Medicaid enrollment	0.992 (0.988-0.997)	.001
Median household income^c^	1.012 (1.009-1.015)	<.001
Total population No.		
<1000	1 [Reference]	
1000-9999	39.565 (32.313-48.445)	<.001
≥10 000	335.570 (269.619-417.653)	<.001
Area		
Urban	1.827 (1.647-2.027)	<.001
Rural	1 [Reference]	
Census division		
New England	1 [Reference]	
Middle Atlantic	0.634 (0.516-0.780)	<.001
East North Central	1.513 (1.246-1.839)	<.001
West North Central	2.851 (2.335-3.481)	<.001
South Atlantic	0.723 (0.592-0.883)	.001
East South Central	1.104 (0.884-1.379)	.38
West South Central	1.877 (1.528-2.305)	<.001
Mountain	0.831 (0.657-1.052)	.12
Pacific	0.597 (0.476-0.749)	<.001
Quartile of older single-occupancy household prevalence^d^		
1: <30.77%	1 [Reference]	
2: 30.77%-36.69%	2.332 (2.087-2.605)	<.001
3: 36.70%-42.39%	4.976 (4.438-5.580)	<.001
4: >42.39%	8.475 (7.444-9.648)	<.001
Constant	0.000 (0.000-0.001)	<.001

Abbreviations: OR, odds ratio; ZCTA, zip code tabulation area.

^a^ Table data based on generalized estimating equation regression, controlled for covariates.

^b^ ORs were calculated per percentage point increase in each variable.

^c^ ORs were calculated per $1000 increase.

^d^ Households with a resident aged at least 65 years or older in which they live alone. Each one-fourth of ZCTAs were one of the categories: quartile 1 (n = 8191), quartile 2 (n = 8148), quartile 3 (n = 8170), and quartile 4 (n = 8169).

The 3 subgroup analyses conducted in ZCTAs with a majority population of White, African American, or Hispanic populations showed differences in the magnitude of the social isolation outcomes (Table 3). The likelihood of having a socially isolated nursing home was approximately 14 times higher in neighborhoods that had a majority of White residents compared with less socially isolated areas (OR, 14.36; 95% CI, 11.31 to 18.23; *P* < .001). In comparison, the likelihood of having a socially isolated nursing home in ZCTAs with a majority population of African American residents or Hispanic residents was approximately 5 to 6 times higher than less isolated areas (African American: OR, 5.40; 95% CI, 4.63 to 6.30; *P* < .001; Hispanic: OR, 6.20; 95% CI, 5.30 to 7.25, *P* < .001). The full results are described in eTable 1, eTable 2, and eTable 3 in the Supplement.

**Table 3.** Odds Ratio of Having Long-term Care Facilities in ZCTA: Subgroup Analyses of ZCTAs Where the Proportion of White, African American, or Hispanic Residents Is Above the Median^a^

Quartile of older single-occupancy household prevalence^b^	Proportion of White residents above the median		Proportion of African American residents above the median		Proportion of Hispanic residents above the median	
	OR (95% CI)	*P* value	OR (95% CI)	*P* value	OR (95% CI)	*P* value
1: <30.77%	1 [Reference]		1 [Reference]		1 [Reference]	
2: 30.77%-36.69%	2.838 (2.265-3.556)	<.001	2.002 (1.760-2.276)	<.001	2.290 (2.015-2.601)	.001
3: 36.70%-42.39%	7.302 (5.835-9.138)	<.001	3.762 (3.293-4.299)	<.001	4.198 (3.667-4.806)	<.001
4: >42.39%	14.358 (11.308-18.231)	<.001	5.402 (4.634-6.296)	<.001	6.195 (5.295-7.248)	<.001

Abbreviations: OR, odds ratio; ZCTA, zip code tabulation area.

^a^ Table data based on generalized estimating equation regression, controlled for covariates.

^b^ Households with a resident aged 65 years or older in which they live alone; estimates were generated from generalized estimating equation regression models controlling for age, sex, race, ethnicity, education, marital status, owner housing, Medicaid enrollment, household income, area, Census division at the ZCTA level.

**Figure 2.**
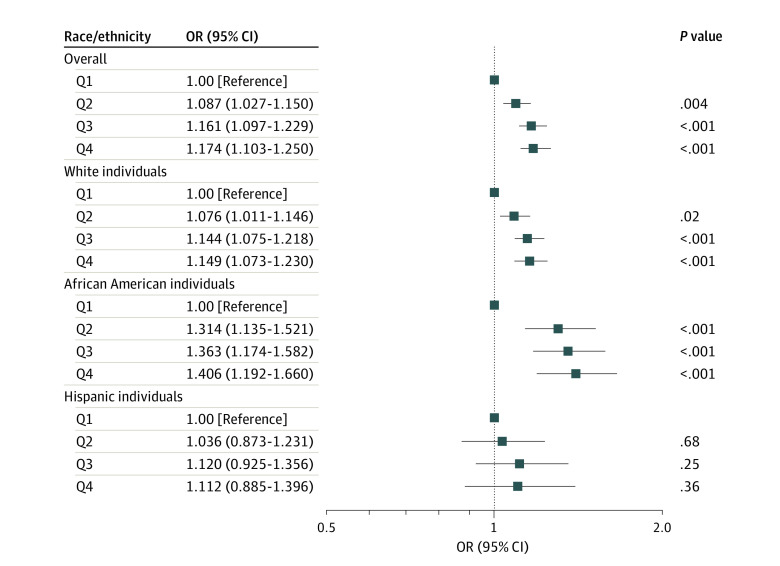
Odds Ratios (ORs) of 30-Day Mortality After Long-term Care Facility Admission by Percentage of Older Single-Occupancy Households in Zip Code Tabulation Area Data calculated from multilevel logistic regression. Q1 indicates quartile 1; Q2, quartile 2; Q3, quartile 3; Q4, quartile 4.

Figure 2 displays the likelihood of 30-day mortality after admission to the LTC facility, controlling for individual, LTC facility, and ZCTA covariates. LTC facilities in more socially isolated neighborhoods were significantly associated with an increased risk of 30-day mortality of residents. The odds of 30-day mortality were approximately 16% to 17% higher in neighborhoods with the 2 highest quartiles of older adults single-occupancy households (quartile 1: OR, 1.17; 95% CI, 1.10-1.25; *P* < .001; quartile 2: OR, 1.16; 95% CI, 1.10-1.23, *P* < .001), and approximately 9% higher in ZCTAs in the third highest quartile (quartile 3: OR, 1.09; 95% CI, 1.03-1.15; *P* < .001) compared with those in the lowest quartile. Subgroup analyses by race and ethnicity identified a higher mortality risk with socially isolated LTC facilities among African American residents compared with White residents. The full results of the multilevel logistic regression models and models with interaction tests are presented in eTable 4, eTable 5, eTable 6, eTable 7, and eTable 8 in the Supplement.

## Discussion

To our knowledge, this is the first study to characterize the geographic locations of LTC facilities within neighborhoods according to the percentage of older adults who live alone. Nationwide, we found that most LTC facilities were located in areas with high social isolation as indicated by the high proportions of older adults living in single-person households in the surrounding ZCTA. This association held across areas with a majority population of African American and Hispanic residents, although the association was stronger in neighborhoods with a majority population of White residents.

Our work suggests that LTC residents may be at increased risk of social isolation that is exacerbated by the location of the facility. This may be important if it creates barriers to connections with friends and family. A review of barriers to family visitations to LTC residents found that travel time to the facility and access to transportation are substantial factors.^25^ Socially isolated LTC facilities may also confer increased risk of negative health risks to their residents, which has been consistently documented in the community setting. Our prior research has found evidence of a shorter survival time based on deaths from all causes in LTC residents who were admitted to facilities in socially isolated neighborhoods at the county level.^14^ In this study, our findings further support that there is an increased risk of short-term 30-day mortality after admission to a LTC facility in socially isolated neighborhoods at the areas level in the same LTC residents. This risk held across White, African American, and Hispanic LTC populations. The increased risk of 30-day mortality could also be an indication of positive placement in the community until the very end of life. Evidence-based solutions for keeping residents of LTC facilities socially engaged are outside the scope of this study, although the telehealth options developed during the pandemic may be a promising platform to keep family and friends connected.

Our analysis of the social isolation of LTC facilities revealed an interesting geographic variation. Most of the socially isolated LTC facilities are in the Midwest section of the US. We have no explanation for this finding, but believe it deserves further investigation.

### Strengths and Limitations

This study had some strengths and limitations. Strengths of our study included its use of a large, nationally representative sample of LTC facilities; a multilevel study design with adjustment for multiple county-level factors; and exploration of differences in associations across race and ethnicity subpopulations.

Limitations of the study relate to the ZCTA-level analyses. Our analyses suggest that the external environments of LTC facilities may influence the social isolation of residents, although we do not test that assumption. Neighborhoods with many older adults living alone may not be a direct measure of social isolation. Similarly, an LTC facility located in an area with large numbers of older adults living alone may not translate to an experience within the facility of social isolation. We lacked individual-level measures of the visit experience of family and friends, which we hypothesize to be associated with the facility’s location. Such measures would be useful in future studies. Additionally, our findings with the risk of mortality are associations that deserve more investigation before causality can be determined.

## Conclusions

This study represents a novel area of inquiry given our growing understanding of the importance of social isolation in older adults who live in LTC facilities. Our work has found that LTCs are often located in socially isolated neighborhoods. This suggests that there may be a need for special attention and strategies to keep LTC residents connected to their family and friends. Such measures could eventually contribute to improved health trajectories in the US population that is increasingly aging and at growing risk of entering LTC facilities.

## Notes

Supplement. **eTable 1.** Odds Ratio (OR) of Having Long-Term Care Facilities in Zip Code Tabulation Area (ZCTA)—Subgroup Analyses of ZCTAs Where the Proportion of Whites is Above the Median **eTable 2. **Odds Ratio (OR) of Having Long-Term Care Facilities in Zip Code Tabulation Area (ZCTA)—Subgroup Analyses of ZCTAs Where the Proportion of African Americans is Above the Median **eTable 3.** Odds Ratio (OR) of Having Long-Term Care Facilities in Zip Code Tabulation Area (ZCTA)—Subgroup Analyses of ZCTAs Where the Proportion of Hispanics is Above the Median **eTable 4.** Odds Ratio (OR) of 30-Day Mortality by Individual, Facility, and Zip Code Tabulation Area (ZCTA)-level Factors (N=730 524) **eTable 5.** Odds Ratio (OR) of 30-Day Mortality: Test of Interactions With Race (N=730 524) **eTable 6.** Odds Ratio (OR) of 30-Day Mortality by Individual, Facility, and Zip Code Tabulation Area (ZCTA)-level Factors—Subgroup Analyses of White **eTable 7.** Odds Ratio (OR) of 30-Day Mortality by Individual, Facility, and Zip Code Tabulation Area (ZCTA)-level Factors—Subgroup Analyses of African Americans **eTable 8.** Odds Ratio (OR) of 30-Day Mortality by Individual, Facility, and Zip Code Tabulation Area (ZCTA)-level Factors—Subgroup Analyses of Hispanics

## References

[1] Abbasi J. Social isolation-the other COVID-19 threat in nursing homes. JAMA. 2020;324(7):619. doi:10.1001/jama.2020.13484 32692848

[2] Nicholson NR. A review of social isolation: an important but underassessed condition in older adults. J Prim Prev. 2012;33(2-3):137-152. doi:10.1007/s10935-012-0271-2 22766606

[3] National Academies of Sciences, Engineering, and Medicine. 2020. Social isolation and loneliness in older adults: Opportunities for the health care system. Washington, DC: The National Academies Press. 32510896

[4] The U.S. Department of Health and Human Services; Administration on Aging, Administration for Community Living. The Administration for Community Living (ACL). 2019 profile of older Americans. Accessed November 17, 2020. https://acl.gov/sites/default/files/Aging%20and%20Disability%20in%20America/2019ProfileOlderAmericans508.pdf

[5] Roberts AW, Ogunwole SU, Blakeslee L, Rabe MA. The Population 65 Years and Older in the United States: 2016, American Community Survey Reports, ACS-38. US Census Bureau; 2018.

[6] Kuiper JS, Zuidersma M, Oude Voshaar RC, . Social relationships and risk of dementia: a systematic review and meta-analysis of longitudinal cohort studies. Ageing Res Rev. 2015;22:39-57. doi:10.1016/j.arr.2015.04.006 25956016

[7] Penninkilampi R, Casey AN, Singh MF, Brodaty H. The association between social engagement, loneliness, and risk of dementia: a systematic review and meta-analysis. J Alzheimers Dis. 2018;66(4):1619-1633. doi:10.3233/JAD-180439 30452410

[8] Campbell-Enns HJ, Campbell M, Rieger KL, Thompson GN, Doupe MB. No other safe care option: nursing home admission as a last resort strategy. Gerontologist. 2020;60(8):1504-1514. doi:10.1093/geront/gnaa077 32589225 PMC7681216

[9] Shaw JG, Farid M, Noel-Miller C, . Social isolation and Medicare spending: among older adults, objective social isolation increases expenditures while loneliness does not. J Aging Health. 2017;29(7):1119-1143. doi:10.1177/0898264317703559 29545676 PMC5847278

[10] Taylor RJ, Taylor HO, Nguyen AW, Chatters LM. Social isolation from family and friends and mental health among African Americans and Black Caribbeans. Am J Orthopsychiatry. 2020;90(4):468-478. doi:10.1037/ort0000448 32309977 PMC8432982

[11] Alcaraz KI, Eddens KS, Blase JL, . Social isolation and mortality in US Black and White men and women. Am J Epidemiol. 2019;188(1):102-109. doi:10.1093/aje/kwy231 30325407 PMC6321805

[12] Pesis-Katz I, Phelps CE, Temkin-Greener H, Spector WD, Veazie P, Mukamel DB. Making difficult decisions: the role of quality of care in choosing a nursing home. Am J Public Health. 2013;103(5):e31-e37. doi:10.2105/AJPH.2013.301243 23488519 PMC3670650

[13] Grabowski DC, Mitchell SL. Family oversight and the quality of nursing home care for residents with advanced dementia. Med Care. 2009;47(5):568-574. doi:10.1097/MLR.0b013e318195fce7 19365290 PMC2709799

[14] Kim D, Park C, Briesacher BA. Socially-isolated neighborhoods and the risk of all-cause mortality among nursing home residents in the United States: a multilevel study. Prev Med Rep. 2020;21:101285. doi:10.1016/j.pmedr.2020.101285 33489720 PMC7804969

[15] Backhaus R, Verbeek H, van Rossum E, Capezuti E, Hamers JP. Nurse staffing impact on quality of care in nursing homes: a systematic review of longitudinal studies. J Am Med Dir Assoc. 2014;15(6):383-393. doi:10.1016/j.jamda.2013.12.080 24529872

[16] Bostick JE, Rantz MJ, Flesner MK, Riggs CJ. Systematic review of studies of staffing and quality in nursing homes. J Am Med Dir Assoc. 2006;7(6):366-376. doi:10.1016/j.jamda.2006.01.024 16843237

[17] Zhang X, Grabowski DC. Nursing home staffing and quality under the nursing home reform act. Gerontologist. 2004;44(1):13-23. doi:10.1093/geront/44.1.13 14978317

[18] U.S. Census Bureau. Decennial census of population and housing: by decade. Accessed October 17, 2019. https://www.census.gov/programs-surveys/decennial-census/decade.2010.html

[19] U.S. Census Bureau. American Community Survey (ACS). Accessed May 6, 2021. https://www.census.gov/programs-surveys/acs

[20] United States Census Bureau / American Fact Finder. American Community Survey. U.S. Census Bureau’s American Community Survey Office. Accessed October 17, 2019. https://factfinder.census.gov/faces/nav/jsf/pages/index.xhtml

[21] Mor V, Angelelli J, Jones R, Roy J, Moore T, Morris J. Inter-rater reliability of nursing home quality indicators in the U.S. BMC Health Serv Res. 2003;3(1):20. doi:10.1186/1472-6963-3-20 14596684 PMC280691

[22] Gambassi G, Landi F, Peng L, . Validity of diagnostic and drug data in standardized nursing home resident assessments: potential for geriatric pharmacoepidemiology. SAGE Study Group. Systematic Assessment of Geriatric drug use via Epidemiology. Med Care. 1998;36(2):167-179. doi:10.1097/00005650-199802000-00006 9475471

[23] Chan E, Procter-Gray E, Churchill L, . Associations among living alone, social support and social activity in older adults. AIMS Public Health. 2020;7(3):521-534. doi:10.3934/publichealth.2020042 32968675 PMC7505797

[24] ArcGIS Hub. ZIP code tabulation areas. Accessed August 6, 2019. https://hub.arcgis.com/datasets/66bc3586afce4fd487f6098a097bc6df_13?geometry=-171.914%2C-76.761%2C171.914%2C76.761

[25] Miller VJ. Investigating barriers to family visitation of nursing home residents: a systematic review. J Gerontol Soc Work. 2019;62(3):261-278. doi:10.1080/01634372.2018.1544957 30412036

