# Supplementary material for: Association of Social Isolation of Long-term Care Facilities in the United States With 30-Day Mortality
Source: JAMA Netw Open. 2021 Jun 16;4(6):e2113361. doi: 10.1001/jamanetworkopen.2021.13361 (PMC8209586; doi:10.1001/jamanetworkopen.2021.13361)
Supplement: Supplement. — eTable 1. Odds Ratio (OR) of Having Long-Term Care Facilities in Zip Code Tabulation Area (ZCTA)—Subgroup Analyses of ZCTAs Where the Proportion of Whites is Above the Median eTable 2. Odds Ratio (OR) of Having Long-Term Care Facilities in Zip Code Tabulation Area (ZCTA)—Subgroup Analyses of ZCTAs Where the Proportion of African Americans is Above the Median eTable 3. Odds Ratio (OR) of Having Long-Term Care Facilities in Zip Code Tabulation Area (ZCTA)—Subgroup Analyses of ZCTAs Where the Proportion of Hispanics is Above the Median eTable 4. Odds Ratio (OR) of 30-Day Mortality by Individual, Facility, and Zip Code Tabulation Area (ZCTA)-level Factors (N=730 524) eTable 5. Odds Ratio (OR) of 30-Day Mortality: Test of Interactions With Race (N=730 524) eTable 6. Odds Ratio (OR) of 30-Day Mortality by Individual, Facility, and Zip Code Tabulation Area (ZCTA)-level Factors—Subgroup Analyses of White eTable 7. Odds Ratio (OR) of 30-Day Mortality by Individual, Facility, and Zip Code Tabulation Area (ZCTA)-level Factors—Subgroup Analyses of African Americans eTable 8. Odds Ratio (OR) of 30-Day Mortality by Individual, Facility, and Zip Code Tabulation Area (ZCTA)-level Factors—Subgroup Analyses of Hispanics [file jamanetwopen-e2113361-s001.pdf]

Supplemental Online Content

Park C, Kim D, Briesacher BA. Association of social isolation of long-term care facilities in the United States with 30-day mortality. *JAMA Netw Open.* 2021;4(6):e2113361. doi:10.1001/jamanetworkopen.2021.13361

eTable 1. Odds Ratio (OR) of Having Long-Term Care Facilities in ZIP Code Tabulation Area (ZCTA)—Subgroup Analyses of ZCTAs Where the Proportion of Whites is Above the Median

eTable 2. Odds Ratio (OR) of Having Long-Term Care Facilities in ZIP Code Tabulation Area (ZCTA)—Subgroup Analyses of ZCTAs Where the Proportion of African Americans is Above the Median

eTable 3. Odds Ratio (OR) of Having Long-Term Care Facilities in ZIP Code Tabulation Area (ZCTA)—Subgroup Analyses of ZCTAs Where the Proportion of Hispanics is Above the Median

eTable 4. Odds Ratio (OR) of 30-Day Mortality by Individual, Facility, and ZIP Code Tabulation Area (ZCTA)-level Factors (N=730 524)

eTable 5. Odds Ratio (OR) of 30-Day Mortality: Test of Interactions With Race (N=730 524)

eTable 6. Odds Ratio (OR) of 30-Day Mortality by Individual, Facility, and ZIP Code Tabulation Area (ZCTA)-level Factors—Subgroup Analyses of White

eTable 7. Odds ratio (OR) of 30-Day Mortality by Individual, Facility, and ZIP Code Tabulation Area (ZCTA)-level Factors—Subgroup Analyses of African Americans

eTable 8. Odds Ratio (OR) of 30-Day Mortality by Individual, Facility, and ZIP Code Tabulation Area (ZCTA)-level Factors—Subgroup Analyses of Hispanics

This supplemental material has been provided by the authors to give readers additional information about their work.

© 2021 Park C et al. *JAMA Network Open.*

eTable 1. Odds ratio (OR) of having long-term care facilities in ZIP Code Tabulation Area (ZCTA) - Subgroup analyses of ZCTAs where the proportion of Whites is above the median

Variable	OR	[95% CI]		P
Age 65+ years (%)	1.057	[1.049,	1.065]	<0.001
Female (%)	1.057	[1.041,	1.073]	<0.001
Education: less than high school (%)	1.002	[0.993,	1.011]	0.718
Education: high school (%)	0.990	[0.983,	0.997]	0.006
Married (%)	0.982	[0.974,	0.990]	<0.001
House owner (%)	0.969	[0.962,	0.976]	<0.001
Medicaid enrollment (%)	1.000	[0.992,	1.008]	0.986
Median household income ($1,000)	1.013	[1.008,	1.019]	<0.001
Total population (n)				
<1,000	Ref.			
1,000-9,999	40.691	[32.081,	51.611]	<0.001
≥10,000	388.235	[293.768,	513.081]	<0.001
Area				
Urban	2.127	[1.706,	2.652]	<0.001
Rural	Ref.			
Census division				
New England	Ref.			
Middle Atlantic	0.709	[0.547,	0.920]	0.010
East North Central	1.979	[1.561,	2.509]	<0.001
West North Central	3.859	[3.027,	4.921]	<0.001
South Atlantic	0.545	[0.401,	0.740]	<0.001
East South Central	1.084	[0.788,	1.491]	0.621
West South Central	1.674	[1.198,	2.339]	0.003
Mountain	1.020	[0.730,	1.423]	0.909
Pacific	0.530	[0.334,	0.841]	0.007
Elderly single-occupancy household (%)				
Q1: <30.77%	Ref.			
Q2: 30.77%-36.69%	2.838	[2.265,	3.556]	<0.001
Q3: 36.70%-42.39%	7.302	[5.835,	9.138]	<0.001
Q4: >42.39%	14.358	[11.308,	18.231]	<0.001
Constant	0.000	[0.000,	0.001]	<0.001

© 2021 Park C et al. *JAMA Network Open.*

eTable 2. Odds ratio (OR) of having long-term care facilities in ZIP Code Tabulation Area (ZCTA) - Subgroup analyses of ZCTAs where the proportion of African Americans is above the median

Variable	OR	[95% CI]		P
Age 65+ years (%)	1.079	[1.070,	1.088]	<0.001
Female (%)	1.027	[1.016,	1.038]	<0.001
Education: less than high school (%)	1.019	[1.013,	1.026]	<0.001
Education: high school (%)	1.004	[0.997,	1.010]	0.253
Married (%)	0.997	[0.991,	1.003]	0.354
House owner (%)	0.986	[0.982,	0.990]	<0.001
Medicaid enrollment (%)	0.990	[0.985,	0.996]	<0.001
Median household income ($1,000)	1.010	[1.006,	1.014]	<0.001
Total population (n)				
<1,000	Ref.			
1,000-9,999	23.962	[17.121,	33.537]	<0.001
≥10,000	185.243	[130.867,	262.213]	<0.001
Area				
Urban	1.510	[1.350,	1.688]	<0.001
Rural	Ref.			
Census division				
New England	Ref.			
Middle Atlantic	0.591	[0.454,	0.770]	<0.001
East North Central	1.260	[0.966,	1.642]	0.088
West North Central	2.051	[1.544,	2.725]	<0.001
South Atlantic	0.608	[0.473,	0.782]	<0.001
East South Central	0.952	[0.723,	1.253]	0.724
West South Central	1.588	[1.221,	2.066]	0.001
Mountain	0.600	[0.431,	0.837]	0.003
Pacific	0.598	[0.451,	0.794]	<0.001
Elderly single-occupancy household (%)				
Q1: <30.77%	Ref.			
Q2: 30.77%-36.69%	2.002	[1.760,	2.276]	<0.001
Q3: 36.70%-42.39%	3.762	[3.293,	4.299]	<0.001
Q4: >42.39%	5.402	[4.634,	6.296]	<0.001
Constant	0.000	[0.000,	0.001]	<0.001

© 2021 Park C et al. *JAMA Network Open.*

eTable 3. Odds ratio (OR) of having long-term care facilities in ZIP Code Tabulation Area (ZCTA) - Subgroup analyses of ZCTAs where the proportion of Hispanics is above the median

Variable	OR	[95% CI]		P
Age 65+ years (%)	1.067	[1.058,	1.076]	<0.001
Female (%)	1.038	[1.025,	1.051]	<0.001
Education: less than high school (%)	1.019	[1.013,	1.026]	<0.001
Education: high school (%)	1.013	[1.007,	1.020]	<0.001
Married (%)	0.997	[0.990,	1.003]	0.285
House owner (%)	0.986	[0.981,	0.990]	<0.001
Medicaid enrollment (%)	0.993	[0.988,	0.999]	0.027
Median household income ($1,000)	1.013	[1.009,	1.017]	<0.001
Total population (n)				
<1,000	Ref.			
1,000-9,999	35.547	[25.241,	50.061]	<0.001
≥10,000	279.303	[195.698,	398.624]	<0.001
Area				
Urban	1.486	[1.323,	1.669]	<0.001
Rural	Ref.			
Census division				
New England	Ref.			
Middle Atlantic	0.644	[0.481,	0.862]	0.003
East North Central	1.373	[1.028,	1.833]	0.032
West North Central	2.864	[2.120,	3.869]	<0.001
South Atlantic	0.769	[0.582,	1.016]	0.064
East South Central	1.112	[0.799,	1.548]	0.528
West South Central	2.131	[1.602,	2.834]	<0.001
Mountain	0.914	[0.670,	1.246]	0.569
Pacific	0.739	[0.551,	0.990]	0.043
Elderly single-occupancy household (%)				
Q1: <30.77%	Ref.			
Q2: 30.77%-36.69%	2.290	[2.015,	2.601]	<0.001
Q3: 36.70%-42.39%	4.198	[3.667,	4.806]	<0.001
Q4: >42.39%	6.195	[5.295,	7.248]	<0.001
Constant	0.000	[0.000,	0.000]	<0.001

© 2021 Park C et al. *JAMA Network Open.*

eTable 4. Odds ratio (OR) of 30-day mortality by individual, facility, and ZIP Code Tabulation Area (ZCTA)-level factors (N=730,524)

		OR	[95% CI]		P
**Individual level**					
Facility stay	Short stayer	Ref.			
	Long stayer	0.013	[0.011,	0.015]	<0.001
Age	≤ 64	Ref.			
	65-79	2.284	[2.179,	2.393]	<0.001
	≥ 80	4.540	[4.327,	4.763]	<0.001
Sex	Male	Ref.			
	Female	0.657	[0.645,	0.670]	<0.001
Race	White, non-Hispanic	Ref.			
	Black, non-Hispanic	0.798	[0.769,	0.828]	<0.001
	Hispanic	0.751	[0.711,	0.793]	<0.001
	Others	0.823	[0.767,	0.883]	<0.001
Marital status	Married	Ref.			
	Never married	1.011	[0.977,	1.047]	0.526
	Widowed	1.113	[1.076,	1.152]	<0.001
	Separated	1.052	[0.960,	1.153]	0.281
	Divorced	0.903	[0.866,	0.942]	<0.001
Medicaid Dual	No	Ref.			
	Yes	1.639	[1.606,	1.673]	<0.001
Depression	No	Ref.			
	Yes	1.758	[1.724,	1.792]	<0.001
RxHCC		2.308	[2.256,	2.362]	<0.001
**Facility level**					
% age 65 and older		0.982	[0.979,	0.984]	<0.001
Age median		1.089	[1.083,	1.096]	<0.001
Sex ratio		0.988	[0.985,	0.992]	<0.001
% White, non-Hispanic		0.997	[0.994,	1.000]	0.027
% Black or African American, non-Hispanic		0.998	[0.995,	1.001]	0.270
% Hispanic		0.999	[0.996,	1.003]	0.697
% married		0.990	[0.987,	0.994]	<0.001
% never married		1.006	[1.003,	1.009]	<0.001
% widowed		0.998	[0.996,	1.001]	0.310
% separated/ divorced		1.004	[1.001,	1.008]	0.007
Number of residents		1.000	[1.000,	1.000]	0.017
% residents on Medicaid		1.012	[1.012,	1.013]	<0.001
Chain Ownership	No	Ref.			
	Yes	1.000	[1.000,	1.001]	0.003
Profit	No	Ref.			
	Yes	1.001	[1.000,	1.001]	<0.001
**ZCTA level**					
% age 65 and older		0.997	[0.992,	1.002]	0.208
Age median		0.991	[0.985,	0.996]	0.001
Sex ratio		1.002	[1.001,	1.004]	<0.001
% owner housing		1.004	[1.003,	1.006]	<0.001
% married		1.006	[1.003,	1.009]	<0.001
% high school or higher		0.994	[0.991,	0.997]	<0.001
Median household income		1.000	[1.000,	1.000]	<0.001
% White alone		1.001	[0.998,	1.003]	0.580
% Black or African American alone		1.000	[0.997,	1.002]	0.960

© 2021 Park C et al. *JAMA Network Open.*

% Hispanic		0.992	[0.990,	0.994]	<0.001
Total population (n)					
<1,000		Ref.			
1,000-9,999		0.906	[0.750,	1.095]	0.306
≥10,000		0.792	[0.656,	0.957]	0.016
Area					
Urban		0.851	[0.823,	0.881]	<0.001
Rural		Ref.			
Census division					
New England		Ref.			
Middle Atlantic		1.165	[1.099,	1.235]	<0.001
East North Central		1.322	[1.248,	1.400]	<0.001
West North Central		1.441	[1.342,	1.548]	<0.001
South Atlantic		1.143	[1.075,	1.216]	<0.001
East South Central		1.257	[1.154,	1.369]	<0.001
West South Central		1.679	[1.567,	1.800]	<0.001
Mountain		1.496	[1.379,	1.623]	<0.001
Pacific		1.366	[1.272,	1.468]	<0.001
Elderly single-occupancy household (%)	Q1: <30.77%	Ref.			
	Q2: 30.77%-36.69%	1.087	[1.027,	1.150]	0.004
	Q3: 36.70%-42.39%	1.161	[1.097,	1.229]	<0.001
	Q4: >42.39%	1.174	[1.103,	1.250]	<0.001
Constant		0.000	[0.000,	0.000]	<0.001

© 2021 Park C et al. *JAMA Network Open.*

eTable 5. Odds ratio (OR) of 30-day mortality: test of interactions with race (N=730,524)

Elderly single-occupancy household (%)	Race	OR	[95% CI]		P
Q1: <30.77%	White	Ref.			
Q2: 30.77%-36.69%	Black	1.216	[1.060,	1.394]	0.005
Q2: 30.77%-36.69%	Hispanic	0.913	[0.788,	1.059]	0.229
Q2: 30.77%-36.69%	Others	0.910	[0.729,	1.135]	0.402
Q3: 36.70%-42.39%	Black	1.147	[1.009,	1.303]	0.036
Q3: 36.70%-42.39%	Hispanic	0.851	[0.738,	0.981]	0.026
Q3: 36.70%-42.39%	Others	0.949	[0.771,	1.169]	0.624
Q4: >42.39%	Black	1.112	[0.982,	1.259]	0.095
Q4: >42.39%	Hispanic	0.905	[0.794,	1.032]	0.136
Q4: >42.39%	Others	0.984	[0.808,	1.199]	0.875

© 2021 Park C et al. *JAMA Network Open.*

eTable 6. Odds ratio (OR) of 30-day mortality by individual, facility, and ZIP Code Tabulation Area (ZCTA)-level factors - Subgroup analyses of White

		OR	[95% CI]		P
**Individual level**					
Facility stay	Short stayer	Ref.			
	Long stayer	0.004	[0.003,	0.005]	<0.001
Age	≤ 64	Ref.			
	65-79	2.312	[2.187,	2.443]	<0.001
	≥ 80	4.599	[4.349,	4.864]	<0.001
Sex	Male	Ref.			
	Female	0.653	[0.640,	0.667]	<0.001
Marital status	Married	Ref.			
	Never married	1.020	[0.980,	1.060]	0.330
	Widowed	1.117	[1.075,	1.162]	<0.001
	Separated	0.981	[0.872,	1.104]	0.754
	Divorced	0.900	[0.858,	0.943]	<0.001
Medicaid Dual	No	Ref.			
	Yes	1.704	[1.666,	1.742]	<0.001
Depression	No	Ref.			
	Yes	1.767	[1.731,	1.804]	<0.001
RxHCC		2.349	[2.288,	2.410]	<0.001
**Facility level**					
% age 65 and older		0.979	[0.977,	0.982]	<0.001
Age median		1.096	[1.089,	1.103]	<0.001
Sex ratio		0.988	[0.984,	0.991]	<0.001
% White, non-Hispanic		0.995	[0.991,	0.999]	0.028
% Black or African American, non-Hispanic		0.998	[0.993,	1.003]	0.406
% Hispanic		0.998	[0.993,	1.004]	0.565
% married		0.992	[0.988,	0.995]	<0.001
% never married		1.007	[1.004,	1.011]	<0.001
% widowed		0.999	[0.996,	1.002]	0.650
% separated/ divorced		1.005	[1.001,	1.009]	0.006
Number of residents		1.000	[0.999,	1.000]	<0.001
% residents on Medicaid		1.011	[1.011,	1.012]	<0.001
Chain Ownership	No	Ref.			
	Yes	1.000	[1.000,	1.001]	0.001
Profit	No	Ref.			
	Yes	1.001	[1.000,	1.001]	0.001
**ZCTA level**					
% age 65 and older		0.998	[0.993,	1.003]	0.446
Age median		0.990	[0.984,	0.995]	<0.001
Sex ratio		1.003	[1.001,	1.004]	<0.001
% owner housing		1.005	[1.003,	1.007]	<0.001
% married		1.006	[1.003,	1.009]	<0.001
% high school or higher		0.994	[0.990,	0.997]	<0.001
Median household income		1.000	[1.000,	1.000]	<0.001
% White alone		1.001	[0.998,	1.004]	0.494
% Black or African American alone		1.000	[0.997,	1.003]	0.844
% Hispanic		0.992	[0.990,	0.994]	<0.001
Total population (n)					
<1,000		Ref.			
1,000-9,999		0.924	[0.758,	1.125]	0.432

© 2021 Park C et al. *JAMA Network Open.*

≥10,000		0.815	[0.670,	0.993]	0.042
Area					
Urban		0.854	[0.824,	0.886]	<0.001
Rural		Ref.			
Census division					
New England		Ref.			
Middle Atlantic		1.181	[1.112,	1.255]	<0.001
East North Central		1.325	[1.249,	1.406]	<0.001
West North Central		1.433	[1.331,	1.543]	<0.001
South Atlantic		1.106	[1.036,	1.181]	0.002
East South Central		1.205	[1.100,	1.320]	<0.001
West South Central		1.659	[1.541,	1.787]	<0.001
Mountain		1.519	[1.395,	1.655]	<0.001
Pacific		1.367	[1.268,	1.475]	<0.001
Elderly single-occupancy household (%)	Q1: <30.77%	Ref.			
	Q2: 30.77%-36.69%	1.076	[1.011,	1.146]	0.021
	Q3: 36.70%-42.39%	1.144	[1.075,	1.218]	<0.001
	Q4: >42.39%	1.149	[1.073,	1.230]	<0.001
Constant		0.000	[0.000,	0.000]	<0.001

© 2021 Park C et al. *JAMA Network Open.*

eTable 7. Odds ratio (OR) of 30-day mortality by individual, facility, and ZIP Code Tabulation Area (ZCTA)-level factors - Subgroup analyses of African Americans

		OR	[95% CI]		P
**Individual level**					
Facility stay	Short stayer	0.000	[0.000,	0.005]	<0.001
	Long stayer				
Age	≤ 64	Ref.			
	65-79	2.395	[2.147,	2.672]	<0.001
	≥ 80	4.588	[4.089,	5.148]	<0.001
Sex	Male	Ref.			
	Female	0.688	[0.645,	0.733]	<0.001
Marital status	Married	Ref.			
	Never married	1.015	[0.923,	1.116]	0.763
	Widowed	1.115	[1.019,	1.221]	0.018
	Separated	1.220	[1.019,	1.462]	0.031
	Divorced	0.916	[0.820,	1.022]	0.115
Medicaid Dual	No	Ref.			
	Yes	1.367	[1.282,	1.458]	<0.001
Depression	No	Ref.			
	Yes	1.601	[1.496,	1.713]	<0.001
RxHCC		2.079	[1.957,	2.209]	<0.001
**Facility level**					
% age 65 and older		0.993	[0.986,	1.000]	0.052
Age median		1.047	[1.029,	1.065]	<0.001
Sex ratio		0.998	[0.988,	1.007]	0.661
% White, non-Hispanic		0.999	[0.987,	1.011]	0.878
% Black or African American, non-Hispanic		0.999	[0.987,	1.011]	0.840
% Hispanic		0.999	[0.986,	1.013]	0.902
% married		0.994	[0.985,	1.003]	0.206
% never married		1.005	[0.997,	1.014]	0.203
% widowed		1.003	[0.994,	1.011]	0.535
% separated/ divorced		1.007	[0.998,	1.016]	0.105
Number of residents		1.001	[1.000,	1.001]	0.009
% residents on Medicaid		1.020	[1.018,	1.022]	<0.001
Chain Ownership	No	Ref.			
	Yes	1.000	[0.999,	1.001]	0.906
Profit	No	Ref.			
	Yes	1.002	[1.001,	1.002]	0.001
**ZCTA level**					
% age 65 and older		0.998	[0.982,	1.014]	0.794
Age median		0.985	[0.969,	1.001]	0.061
Sex ratio		1.003	[1.000,	1.006]	0.094
% owner housing		1.005	[1.001,	1.009]	0.015
% married		1.013	[1.005,	1.021]	0.001
% high school or higher		0.993	[0.986,	1.000]	0.067
Median household income		1.000	[1.000,	1.000]	0.011
% White alone		0.996	[0.991,	1.002]	0.229
% Black or African American alone		0.997	[0.991,	1.004]	0.400
% Hispanic		0.990	[0.984,	0.995]	<0.001
Total population (n)					
<1,000		Ref.			
1,000-9,999		0.832	[0.443,	1.561]	0.566

© 2021 Park C et al. *JAMA Network Open.*

≥10,000		0.731	[0.391,	1.367]	0.327
Area					
Urban		0.910	[0.823,	1.005]	0.064
Rural		Ref.			
Census division					
New England		Ref.			
Middle Atlantic		1.035	[0.829,	1.292]	0.763
East North Central		1.198	[0.961,	1.495]	0.109
West North Central		1.394	[1.064,	1.825]	0.016
South Atlantic		1.199	[0.964,	1.490]	0.103
East South Central		1.431	[1.104,	1.855]	0.007
West South Central		1.650	[1.303,	2.090]	<0.001
Mountain		1.039	[0.714,	1.513]	0.840
Pacific		1.530	[1.171,	1.999]	0.002
Elderly single-occupancy household (%)	Q1: <30.77%	Ref.			
	Q2: 30.77%-36.69%	1.314	[1.135,	1.521]	<0.001
	Q3: 36.70%-42.39%	1.363	[1.174,	1.582]	<0.001
	Q4: >42.39%	1.406	[1.192,	1.660]	<0.001
Constant		0.000	[0.000,	0.001]	<0.001

© 2021 Park C et al. *JAMA Network Open.*

eTable 8. Odds ratio (OR) of 30-day mortality by individual, facility, and ZIP Code Tabulation Area (ZCTA)-level factors - Subgroup analyses of Hispanics

		OR	[95% CI]		P
**Individual level**					
Facility stay	Short stayer	Ref.			
	Long stayer	1.000			
Age	≤ 64	Ref.			
	65-79	1.766	[1.460,	2.135]	<0.001
	≥ 80	3.588	[2.959,	4.351]	<0.001
Sex	Male	Ref.			
	Female	0.685	[0.621,	0.755]	<0.001
Marital status	Married	Ref.			
	Never married	0.910	[0.780,	1.061]	0.229
	Widowed	1.075	[0.920,	1.255]	0.365
	Separated	1.131	[0.844,	1.516]	0.411
	Divorced	0.905	[0.752,	1.090]	0.294
Medicaid Dual	No	Ref.			
	Yes	1.171	[1.053,	1.303]	0.004
Depression	No	Ref.			
	Yes	1.969	[1.778,	2.181]	<0.001
RxHCC		2.426	[2.189,	2.688]	<0.001
**Facility level**					
% age 65 and older		0.999	[0.987,	1.011]	0.891
Age median		1.071	[1.041,	1.102]	<0.001
Sex ratio		0.846	[0.762,	0.940]	0.002
% White, non-Hispanic		1.004	[0.993,	1.015]	0.519
% Black or African American, non-Hispanic		1.003	[0.991,	1.015]	0.612
% Hispanic		1.003	[0.991,	1.015]	0.654
% married		0.982	[0.970,	0.993]	0.001
% never married		1.000	[0.990,	1.011]	0.94
% widowed		0.993	[0.983,	1.003]	0.172
% separated/ divorced		0.997	[0.985,	1.008]	0.590
Number of residents		1.000	[0.999,	1.001]	0.969
% residents on Medicaid		1.016	[1.013,	1.020]	<0.001
Chain Ownership	No	Ref.			
	Yes	1.000	[0.999,	1.001]	0.578
Profit	No	Ref.			
	Yes	1.000	[0.998,	1.001]	0.863
**ZCTA level**					
% age 65 and older		0.963	[0.938,	0.987]	0.003
Age median		1.026	[1.000,	1.052]	0.051
Sex ratio		0.996	[0.990,	1.002]	0.172
% owner housing		1.002	[0.996,	1.007]	0.549
% married		1.008	[0.995,	1.020]	0.230
% high school or higher		0.997	[0.988,	1.007]	0.603
Median household income		1.000	[1.000,	1.000]	0.002
% White alone		0.996	[0.990,	1.003]	0.258
% Black or African American alone		0.991	[0.982,	0.999]	0.034
% Hispanic		0.994	[0.988,	1.000]	0.053
Total population (n)					
<1,000		Ref.			
1,000-9,999		1.073	[0.130,	8.832]	0.947

© 2021 Park C et al. *JAMA Network Open.*

≥10,000		0.985	[0.121,	8.025]	0.989
Area					
Urban		0.847	[0.733,	0.979]	0.025
Rural		Ref.			
Census division					
New England		Ref.			
Middle Atlantic		1.101	[0.824,	1.472]	0.515
East North Central		1.510	[1.121,	2.033]	0.007
West North Central		1.719	[1.044,	2.833]	0.033
South Atlantic		1.249	[0.924,	1.688]	0.148
East South Central		1.666	[0.667,	4.163]	0.275
West South Central		1.899	[1.400,	2.575]	<0.001
Mountain		1.365	[0.992,	1.878]	0.056
Pacific		1.381	[1.030,	1.854]	0.031
Elderly single-occupancy household (%)	Q1: <30.77%	Ref.			
	Q2: 30.77%-36.69%	1.036	[0.873,	1.231]	0.684
	Q3: 36.70%-42.39%	1.120	[0.925,	1.356]	0.247
	Q4: >42.39%	1.112	[0.885,	1.396]	0.362
Constant		0.000	[0.000,	0.001]	<0.001

© 2021 Park C et al. *JAMA Network Open.*
